# Tribology of Copper Metal Matrix Composites Reinforced with Fluorinated Graphene Oxide Nanosheets: Implications for Solid Lubricants in Mechanical Switches

**DOI:** 10.1021/acsanm.3c00399

**Published:** 2023-05-10

**Authors:** Nicky Savjani, Vicente Orts Mercadillo, Darren Hodgeman, George Paterakis, Yubao Deng, Cristina Vallés, George Anagnostopoulos, Costas Galiotis, Mark A. Bissett, Ian A. Kinloch

**Affiliations:** † Department of Materials, Henry Royce Institute and National Graphene Institute, The University of Manchester, Oxford Road, Westminster M13 9PL, U.K.; ‡ Carbon Science Center of Excellence, Morgan Advanced Materials and Technology, Inc., 310 Innovation Boulevard, Technology Center, Suite 250, University Park, Pennsylvania 16803, United States.; § Foundation for Research and Technology Hellas, Institute for Chemical Engineering Sciences, Stadiou Street, Platani, Patras GR26504, Greece; ∥ Department of Chemical Engineering, University of Patras, Patras 26504, Greece

**Keywords:** fluorographene, copper metal matrix composite, solid lubrication, corrosion resistance, tribology, tribochemistry

## Abstract

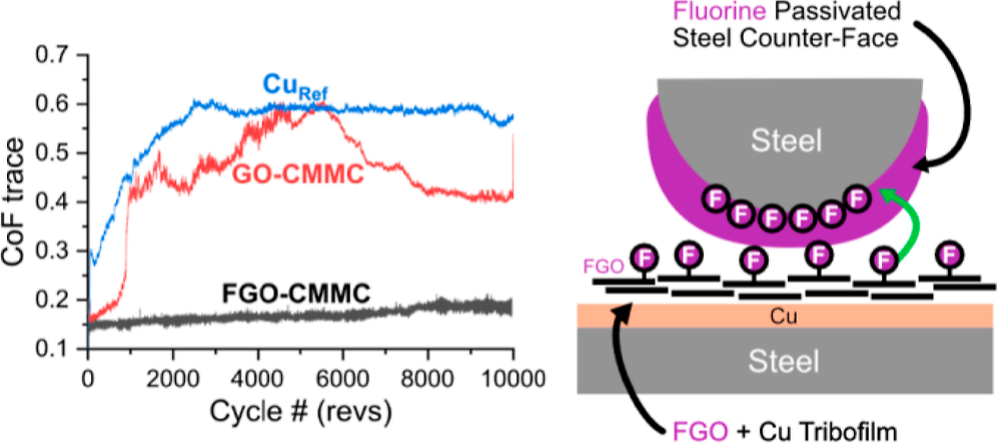

The potential for the use of copper coatings on steel switching mechanisms is abundant owing to the high conductivities and corrosion resistance that they impart on the engineered assemblies. However, applications of these coatings on such moving parts are limited due to their poor tribological properties; tendencies to generate high friction and susceptibility to degradative wear. In this study, we have fabricated a fluorinated graphene oxide–copper metal matrix composite (FGO-CMMC) on an AISI 52100 bearing steel substrate by a simple electrodeposition process in water. The FGO-CMMC coatings exhibited excellent lubrication performance under pin-on-disk (PoD) tribological sliding at 1N load, which reduced CoF by 63 and 69%, compared to the GO-CMMC and pure copper coatings that were also prepared. Furthermore, FGO-CMMC achieved low friction and low wear at higher sliding loads. The lubrication enhancement of the FGO-CMMCs is attributed to the tribochemical reaction of FGO with the AISI 52100 steel counterface initiated by the sliding load. The formation of an asymmetric tribofilm structure on the sliding track is critical; the performance of the FGO/Cu tribofilm formed in the track is boosted by the continued fluorination of the counterface surface during PoD sliding, passivating the tribosystem from adhesion-driven breakdown. The FGO-CMMC and GO-CMMC coatings also provide increased corrosion protection reaching 94.2 and 91.6% compared to the bare steel substrate, allowing for the preservation of the long-term low-friction performance of the coating. Other influences include the improved interlaminar shear strength of the FGO-containing composite. The excellent lubrication performance of the copper matrix composite coatings facilitated by FGO incorporation makes it a promising solid lubricant candidate for use in mechanical engineering applications.

## 1 Introduction

Steel is the material of choice for switching mechanisms, contacts, and mechanical assemblies used under special environments and extreme working conditions.^1^ However, to achieve a suitable performance of steel alloys within the application domain, with the ability to operate many thousands of times within a low load assembly (Herzian contact pressures <500 MPa), two key limitations are needed to be overcome:^2^ (1) Mechanical friction is the ubiquitous force that resists motion when two mutually contacting surfaces are moved relative to each other, due to sliding and rolling wear, fatigue load, and fretting damage over its operational lifetime,^3,4^ seriously reducing the service life of components.^5^ (2) Metal corrosion, which is triggered by environmental factors, such as the presence of oxygen,^6^ water,^7^ ultraviolet (UV),^8^ and electrolytes,^9^ can adversely affect the infrastructure of mechanical assemblies.^10^ Reports indicate that approximately 23^11^ and 20%^12,13^ of the world’s energy consumption is lost to friction and corrosion, respectively. Applying coatings to steel contacts can provide a route to minimize, protect, and prevent the effects of friction, wear, and corrosion within switch assemblies, which would reduce failures, in addition to improving energy efficiencies and lowering their environmental impact.^14−19^

Copper-coating of mechanical components and surfaces is commonplace in industry^20^ due to the coatings’ excellent chemical and physical properties imparted onto the surface of a component. These include its high thermoelectrical conductivities, corrosion resistance, and antimicrobial properties.^21−24^ However, the application of copper coatings within contact mechanisms is limited due to its poor wear resistance under applied load.^25^ Therefore, improving the wear resistance of copper has become a key issue for use in high-performance mechanical components.

Lamellar materials such as graphite have been extensively used as solid lubricants over the last 60 years.^26^ As a popular solid lubricant, graphite possesses many attractive features; excellent mechanical properties, low-friction coefficient from its low anisotropic shear strength, and good wear resistance.^27^ Its two-dimensional analogue, graphene,^28^ has shown significant promise in ultralow friction in both nanoscale and microscale lubrication,^29−32^ supported by *in silico* measurements.^33−35^ The materials’ extremely high strength, low shear resistance, relatively low cost, and comparably high thermal conduction and dissipation performances are attributed to the materials’ 2D-lamellar structures.^36−38^ Due to its unique structure and chemical properties, graphene is essentially impermeable to most chemicals and gases.^39^ Thus, it can be considered as a corrosion barrier providing high protection even in coatings of a few nanometer thicknesses.^40^

More recently, a fluorine-functionalized derivative, fluorographene, has been explored as a potential candidate for macroscale low-friction lubrication.^41,42^ Theoretically, the chemical and physical structures of fluorographenes should provide a route to ultralow friction. However, this has not been borne out experimentally^41,43−45^ as increased defect generation during fluorination increased the nanomaterial shear strengths in macroscale sliding.

The development of advanced self-lubricating materials and coatings is a potentially promising approach for improving frictional, protection, and wear properties in surface engineering^46,47^ and is a target to replace grease-, oil-,^48^ and noble-metal^49,50^-lubricated systems within high-load, low-temperature mechanisms. Copper metal matrix composites (CMMCs)^51^ could serve as the frictional material in electrical sliding contacts, such as brushes, bearing bushings, and contact wires,^52−54^ due to excellent thermal and electrical properties, as well as to expand into new horizons, including incorporation into high-speed transport and aerospace mechanisms.^55^ The inclusion of graphene into CMMCs^52−54,56−58^ has been proposed to enhance the desired mechanical functionalities of the copper, acting as second-phase reinforcement to improve hardness and fracture toughness by blocking dislocation motion^59−63^ and to generate a lubricating medium upon sliding contact of the composite.^64,65^ Previous studies^62^ have shown that 0.025 wt % reduced graphene oxide in sintered CMMC improves yield stresses by ∼ 100%. 1.1 wt % rGO inclusion into an electrodeposited CMMC coating improves the surface microhardness by ∼ 30%,^64^ while a CMMC coating with 0.83 wt % GO protects against corrosion by up to 94.9%.^66^ Furthermore, low graphitic loadings (sub-10 wt %) into copper films greatly diminish frictional losses under low contact pressure nano-and macrotribological testing.^38,67,68^ Fluorographenes have been utilized in oil-based^69−73^ and polymer-based^74−76^ lubricating formulations. However, the incorporation of fluorographenes into metal composites has scarcely been explored.^77,78^

It is still a challenge to homogeneously disperse graphenes in metal matrices with robust interfacial bonding as the chemical inertness of both graphene and copper limits their interfacial compatibility within the composite.^79^ Other factors include their varying wettabilities^58^ and thermal expansion coefficients^59^ causing structural defects and the low stability of graphene dispersion within traditional, aqueous copper-based plating solutions inhibiting uniform distribution of the nanomaterials within the matrix.^38,80^ By promoting good interfacial interactions between the components of CMMCs, it is possible to improve its frictional performance. Therefore, improving the dispersibility and interaction parameters between graphene and copper is key to obtaining high tribological performance. This has been explored recently by employing functionalized graphenes (such as graphene oxides^38,59,81,82^); however, the subsequent tribo-induced chemical reactions on the functionalized composites rarely examine the role of the dopant moieties within sliding contact.

Although some mechanisms such as copper-grain refinement and formation of tribofilms were proposed to explain the tribological behavior of graphene–metal matrix composites,^83^ the tribo-induced chemical reactions on the friction surfaces during a friction process are still unclear. In particular, the tribochemical information of graphene is needed to gain insights into the tribological mechanisms of the composite as a whole.

In this study, CMMCs containing fluorinated graphene oxide (FGO) were uniformly deposited onto AISI 52100 bearing steel substrates by electrodeposition in water. These fluorinated graphene oxide–copper metal matrix composites (FGO-CMMCs) exhibited excellent macroscale lubrication as well as enhancing the protection of the metal surface from corrosion, compared to graphene oxide (GO) containing CMMCs and pure copper coatings. The mechanism for the enhanced lubrication performance was also studied in detail.

## 2 Experimental Section

### 2.1 Materials

A GO slurry was received from The Sixth Element (Changzhou) Materials Technology Co. Ltd. The slurry was frozen overnight before freeze-drying to produce a dehydrated powder, which was blended and sieved to <50 μm. FGO was prepared using plasma-fluorination. The Haydale HT-60 lab scale reactor was used to process a 25 g batch of material in a CF_4_ plasma, at low temperatures (<100 °C) and pressures (<1 mbar). A detailed characterization of the GO and FGO powders is provided in ESI (Figures S1–S3, Table S1).

The components of the nickel-Watts (nickel sulfate, nickel chloride, and boric acid) and copper [etidronic acid, copper(II) carbonate hydroxide, potassium pyrophosphate, sulfamic acid, potassium hydroxide, and potassium carbamate] plating solutions were purchased from Sigma-Aldrich. All chemicals were used as supplied.

The nickel-Watts plating solution was produced, as described elsewhere.^84^ The alkaline copper-plating solution was modified from the cited recipe:^85^ for a liter of the solution, basic copper carbonate (30 g) was initially dissolved in etidronic acid (65 wt %; 150 g) and made up to 600 mL with deionized water, before the addition of sulfamic acid (50 g) and potassium pyrophosphate (100 g). The solution was raised to pH 6 by the slow addition of potassium hydroxide (∼70 g); gelation occurs at pH 5, which redissolves at higher pH. Finally, potassium carbonate was added until a pH of 9 is obtained (∼100 g).

FGO-copper and GO-copper plating dispersions were prepared by loading the basic copper plating solution with FGO or GO powders to achieve a 1 mg/mL dispersion, followed by ultrasonication for 2 h (Elmasonic P70H, 37 kHz amplitude, 100% power) and storage. Before use, the dispersions were agitated by sonication for 15 min.

SKF LS-1528 (AISI 52100, 28 mm OD, 15 mm ID, 2.75 mm thick) and LS-2542 (AISI 52100, 42 mm OD, 25 mm ID, 3.00 mm thick) raceway washers were purchased from shop.Eriks.co.uk. 8 mm diameter 52100 ball bearings (Grade 10: *R*_a_ ≤25 nm) were purchased from Simplybearings.co.uk. Both the 52100 washers and ball bearings are used as supplied. The 52100 washers and ball bearings were cleaned before use by successive ultrasonic cleaning steps in isopropanol (twice) and acetone followed by freeboard drying. Once cleaned, the materials were seal-packed in low density polyethylene (LDPE) bags until required. For thickness determination of a given coating, eight areas of the substrate were masked with latex before deposition: four along the inner edge and four on the outer edge of the 52100 washer (Figure S4).

### 2.2 Methods

**Figure 1:**
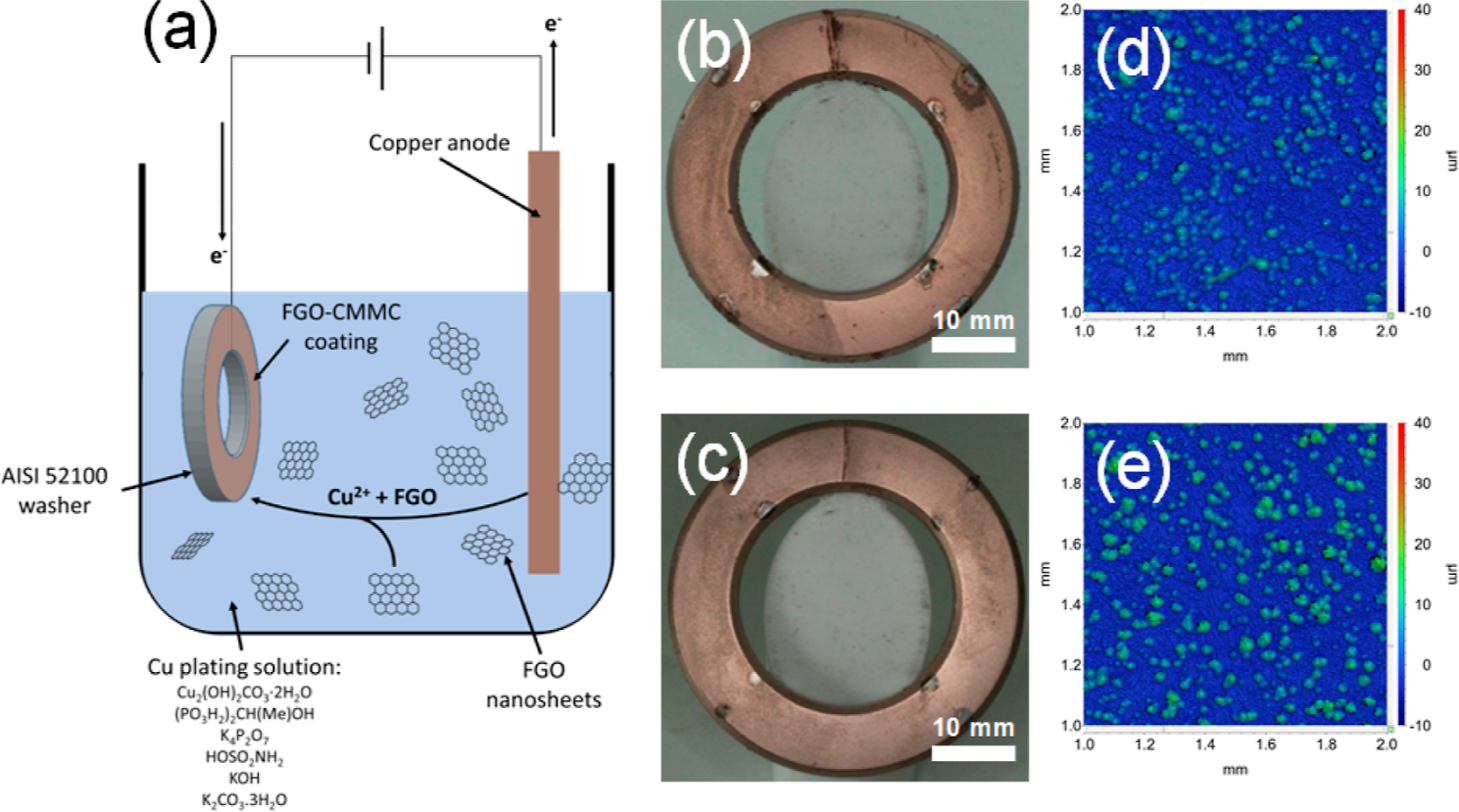
Preparation of the FGO-CMMC coatings on AISI 52100 steel. (a) Schematic of the electrodeposition process. (b,c) Photographs of the coatings prepared at a current density of (b) 0.25 A/dm^2^ [FGO-CMMC_(0.25CD)_] and (c) 0.5 A/dm^2^ (FGO-CMMC_(0.5CD)_). (d,e) WL interferometry topographs of the coatings from (b) and (c).

Electroplating of the FGO-CMMC, GO-CMMC, and Cu_Ref_ coatings onto the 52100 substrates was achieved using an electroplating assembly (Figure 1a). The assembly consisted of a 400 mL tall form borosilicate glass beaker, with 200 mL of plating solution and a magnetic stirrer to ensure homogeneous mixing during electroplating. Four Cu sheets (approx. 20 × 1 × 200 mm) were evenly spaced around the inside perimeter of the beaker and connected to act as the anode. The cathode was made from a masked AISI 52100 washer, suspended in the plating solution using Cu wire. The current density was controlled using a current-limited power supply (Agilent E3631A). To assist with uniform FGO-CMMC and GO-CMMC co-plating, a 100 nm nickel flash layer was deposited (Watts solution, 1 A/dm^2^, 30 s) onto the AISI 52100 washer before Cu-plating. After washing the Ni-flashed 52100 substrate with water and isopropanol, it was plated in the respective plating dispersions/solutions for FGO-CMMC, GO-CMMC, and Cu_Ref_ deposition experiments. The current density and deposition time were adjusted to achieve ∼20 μm thick coatings on the washer (0.25 A/dm^2^ for 6 h, or 3 h at 0.5 A/dm^2^). After completing a deposition, each coating was stored with silica gel to inhibit oxidation.

White light (WL) Interferometry measurements for FGO-CMMC, GO-CMMC, and Cu_Ref_ coatings were undertaken using a Bruker Contour-GT white-light profilometer equipped with a 2.5× optical lens. The reported thickness of a given CMMC is based on the average thickness measured across each of the paired masked areas (Figure S4; Red markings). All surface roughness (*R*_a_) measurements were also obtained by WL profilometry, from a ∼9 mm^2^ area of the coating located at the same position on each sample (Figure S4; black markings).

The surfaces of the FGO-CMMC, GO-CMMC, and Cu_Ref_ coatings were observed by scanning electron microscopy (SEM, Quanta-250 FEG-SEM) at 20 keV along with energy-dispersive spectroscopy (EDX, Xmax 80, Oxford instruments). The focal length for imaging and analysis was fixed at 10 mm.

Grazing incidence X-ray diffraction (XRD) studies were performed using a Philips X’Pert–MPD theta–theta diffractometer (400 mm diameter) with a PW1711 (Proportional) point detector in Bragg–Brentano geometry employing a copper line focus X-ray tube with Ni k_β_ absorber (0.02 mm; Kβ = 1.392250 Å) Kα radiation (Kα_1_ = 1.540598 Å, Kα_2_ = 1.544426 Å, Kα ratio 0.5, and Kα_av_ = 1.541874 Å). Incident beam Soller slit of 0.04 rad, incident beam mask of 10 mm, programmable automated divergence slit giving a constant illuminated length of 10.0 mm, programmable anti-scatter slit observed length of 10.0 mm, receiving Soller slit of 0.04 rad, parallel plate collimator (0.27°), and diffracted beam curved graphite monochromator (002) were used. Data collection from 3 to 90° 2θ scan at 0.02° step size and 4 s/step was undertaken.

Raman spectroscopy was carried out in a Renishaw inVia Raman spectrometer using a laser excitation wavelength of 514 nm at 100x magnification, operating at approx. 1 mW. The spectra were acquired through a spectrum window of 250–3500 cm^–1^. In all experiments, the 52100 substrate does not contribute to any signals within the observed Raman spectral window.

Microindentation experiments were carried out using a CSM Micro Indentation tester equipped with a Vickers diamond tip. All indentations were carried out with a 5N load for direct comparison.

The performance of the FGO- and GO-CMMC coating in the corrosion protection was evaluated electrochemically through potentiostatic and potentiodynamic measurements under a 3.5% NaCl environment. The corrosion resistance of the samples was estimated in an electrochemical cell consisting of a silver/silver chloride (Ag/AgCl, KCl 3 M) electrode and a platinum sheet as reference and counter electrodes, respectively.^86^ The examined samples were sealed on the base of the cell to make corrosion possible only on the coated side (an active area of 1 cm^2^). The corrosion measurements were performed on each sample, first by recording the open-circuit potential (OCP) during a stabilization period of 1 h, followed by potentiodynamic polarization measurements at a scan rate of 0.3 mV/s within a scan range of ±250 mV vs OCP.

Reciprocating pin-on-disk (PoD) sliding tests were performed on a Bruker UMT Tribolab tribometer with a 1–100N load cell installed and operated at ambient conditions (20 °C and approx. 45% RH). The data sample rate of 1000 Hz was selected. The CoF trace (μ) is obtained by first processing the applied load (*F*_*z*_) and frictional force (*F*_*x*_) traces to generate an *F*_*x*_/*F*_*z*_ trace. To remove datapoints attributed to static (and quasi-static) friction modes, the value for μ for each stroke (= 0.5 Hz) is taken from the average of the middle 50% of the *F*_*x*_/*F*_*z*_ trace as this represents the dynamic friction phase of the sliding tests.

## 3 Results and Discussion

### 3.1 Coating Fabrication and Characterization

Three sets of coatings were prepared by electrodeposition of the respective graphene-copper plating dispersions onto AISI 52100 steel washers (Figure 1a); FGO- and GO-containing copper coatings onto AISI 52100 (FGO-CMMC and GO-CMMC), alongside graphene-free copper coatings (Cu_Ref_) for comparison. For each set of coatings, the effect of deposition power was further studied by preparing coatings at two current densities (0.25 and 0.5 A/dm^2^).

**Figure 2:**
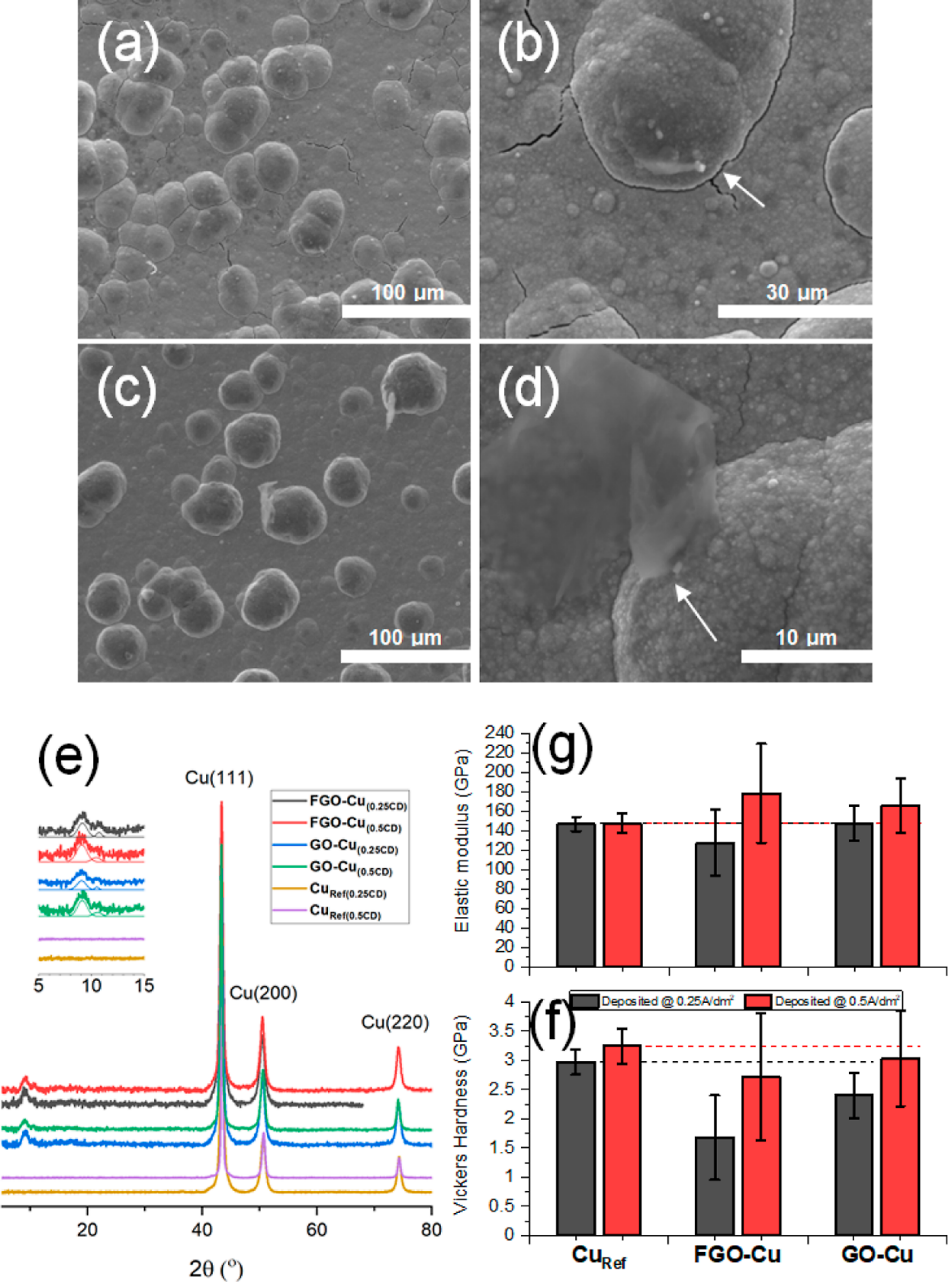
Physical and spectroscopic characterization of CMMC coatings on AISI 52100 steel: scanning electron microscopy (SEM) images of the coatings: (a) FGO-CMMC_(0.25CD)_, (c) FGO-CMMC_(0.5CD)_, (b,d) are magnified sections of (a,c), highlighting the capture of FGO within the coating structure. (e) X-ray diffractograms of all FGO-CMMC, GO-CMMC, and Cu_Ref_ coatings; inset expands on the FGO and GO peaks observed within the composite coatings. (f) Vickers microhardness and (g) reduced elastic moduli of the FGO-CMMC, GO-CMMC, and Cu_Ref_ coatings, and values were obtained from an average of 49 microindentations.

FGO-CMMC coatings were uniformly deposited onto steel washers (Figure 1b,c). Thicknesses of the FGO-CMMCs prepared at 0.25 and 0.5 A/dm^2^ were 18.4 and 18.3 μm, respectively, close to the 20 μm thicknesses targeted for the project (Figure S5). The equivalent GO-CMMC and CuRef coatings were also produced; their topographical features are described in detail in the ESI. The surface morphology of the FGO-CMMC, GO-CMMC, and Cu_Ref_ coatings was assessed by WL interferometry and SEM. The equivalent GO-CMMC and Cu_Ref_ coatings were also produced; their topographical features are ascribed in detail in the ESI (Figure S6). The surface morphology of the FGO-CMMC, GO-CMMC, and CuRef coatings were assessed by WL interferometry and SEM. It can be observed that FGO-CMMC and GO-CMMC coatings have a similar topography across the whole substrate; the surface of the coating is mostly level but is perturbed by a significant number of additional growths that protrude above the level-plated surface (Figure 1d,e). The sizes of these growths range from a few microns to up to ∼60 μm. Often, FGO and GO nanosheets are found embedded inside these growths protruding from their core, as identified by SEM imaging (Figure 2a–d, Supporting Information S6i,j,m,n). All nanosheets identified within the composites showed no copper nanoparticle growth on their surface, suggesting that the graphitic sheets are anchored into the Cu coating only by edge interactions before further coating growth captures the nanosheets within the copper heterophase. The surfaces of both FGO-CMMC and GO-CMMC coatings contain microfractures (Figure 2b,d). These cracks arise from the strain-fracturing of grain boundaries between dislocation growths of differing orientations after deposition. It appears from both mechanical and tribological data presented later that these fractures are superficial and do not impact the integrity of the coating during tribological testing.

WL interferometry topographs show that FGO-CMMC coatings show higher coverage of these growths on the plated surface (∼40–50% in a 3.75 mm^2^ area vs ∼10% for GO-CMMCs); however, the footprints of these growths on the GO-CMMC coatings are significantly larger (Table S3). The combination of the higher surface area coverage and smaller dislocation growth sizes observed on FGO-CMMC coatings may suggest that FGO co-deposits without aggregation, resulting in a more uniform distribution of the FGO nanosheets within the FGO-CMMC coating structure. This is in contrast to GO-CMMCs, which show evidence of a lower concentration of larger volume dislocation co-deposits which is a result of GO nanosheet aggregation before deposition. Due to the presence of these dislocation growths, the *R*_a_ values of both sets of FGO-CMMC and GO-CMMC coatings have increased to >1 μm (*c.f.* <300 nm for Cu_Ref_).

XRD studies reveal information on the microcrystalline nature of the CMMCs produced by co-deposition with graphene (Figure 2e). The 0.25 and 0.5 A/dm^2^ coatings are compared to their equivalent Cu_Ref_ coatings. All coatings show a pattern consistent with pure copper (fcc) with no evidence of lattice strain induced by graphene incorporation.^87^ The Ni flash layer is absent due to its thinness and amorphous nature. The FGO-CMMC and GO-CMMC coatings display a small diffraction peak at ∼9° relating to the (001) diffraction of (F)GO nanosheets within the coating. This can be further deconvoluted into two peaks, as shown in the figure inset, indicative of two crystallographic phases: The dominant peak (at 9.3 and 9.1°) corresponds to restacked FGO and GO with an increased interlayer spacing (9.7 and 9.3 Å), while the weaker peak (at 10.9 and 10.8°) corresponds to more tightly packed native FGO and GO (8.2 and 8.0 Å). The latter values are representative of the (001)-stacking of FGO and GO powders characterized in the ESI (Figure S1). In all cases, the relative intensity of the graphitic peaks to the Cu pattern is consistent, suggestive of a similar graphitic concentration within the CMMCs.

The co-deposition of (F)GO into CMMCs has a significant effect on their mechanical properties (Figure 2f,g). FGO-CMMC_(0.25CD)_ shows the most pronounced effects as its hardness (1.67 GPa) is considerably lower than that for both Cu_Ref(0.25CD)_ and GO-CMMC_(0.25CD)_ (2.97 and 2.39 GPa). This accounts for ∼43 and 18% reduction in hardness for the copper coating upon FGO and GO incorporation when deposited at 0.25 A/dm^2^. This results in a coating that has lower elasticity and higher ductility—desired properties in solid lubrication systems facilitated by soft metals.^88^ Less significant reductions in *H* values were reported when deposited at 0.5 A/dm^2^, with values of 2.72, 3.03, and 3.24 GPa for FGO-CMMC_(0.5CD)_, GO-CMMC_(0.5CD)_, and Cu_Ref(0.5CD)_ coatings, confirming a 16 and 7% reduction in hardness of the copper coatings by FGO and GO incorporation. These findings are in contrast to the literature, which shows that bulk graphene–copper matrix composites can achieve increases in Vickers Hardnesses of up to 300% compared to pure copper.^89−91^ However, these bulk systems are produced in methods where graphene contributes to the grain refinement of the copper composite; the incorporation of graphene during the sintering prevents Cu-grain growth and, therefore, promotes increased resistance to mechanical deformation.^89^ The low temperature and slow growth rate in the electrodeposition conditions applied for FGO-CMMC and GO-CMMC production are not limited by such effects, providing a route to lowering the hardnesses of the composites, rather than increasing them. The elastic moduli of all FGO-CMMC and GO-CMMC are (within error) consistent with observed values for their Cu_Ref_ counterparts, albeit with a higher degree of deviation as a cause of the rougher surfaces from graphene incorporation.

### 3.2 Mechanism for FGO-CMMC and GO-CMMC Co-deposition

**Figure 3:**
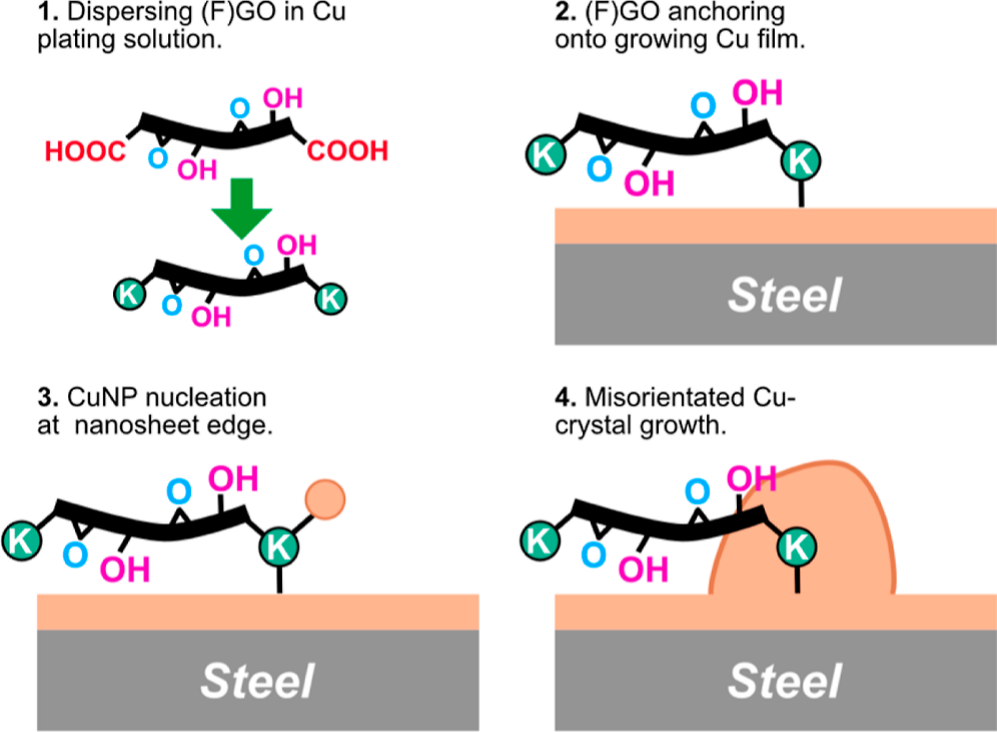
Postulated mechanism for the co-deposition of FGO-CMMCs and GO-CMMCs on AISI 52100 steel.

A detailed chemical analysis of the FGO and GO powders and filtrates (post-dispersion in the copper plating solution) is provided in the ESI. Alongside the information obtained on the coatings, a mechanism for the deposition of FGO-CMMCs and GO-CMMCs onto AISI 52100 steel can be tentatively proposed (Figure 3). The example below focuses on the use of GO within the mechanism for clarity but can also be interpreted for FGO inclusion. Evidence from XRD, attenuated total reflectance-Fourier transform infrared (ATR-FTIR) spectroscopy, and X-ray photoelectron spectroscopy (XPS) shows that in the production of the plating dispersion, the carboxylic acid groups on (F)GO nanosheets are functionalized by a K^+^ salt in the solution, resulting in an edge-functionalized nanosheet. The nature of the ion functionalization is not clear, but XPS evidence suggests that a form of (K^+^)_x_(CO_3_)_x_ is present. The functionalization of the graphitic nanosheets also aids in stabilizing the plating dispersion to aggregation. During deposition, the metallic moieties on the functionalized graphenes anchor to the growing copper film by edge interactions. With continued growth, the GO nanosheet binds to the growing copper film in a passive manner. There is no further chemical activation of the nanosheet surface (namely, at the alkoxy/epoxy sites), inhibiting any CuNP nucleation on its surface. The binding interface between Cu and the nanosheets may induce a nucleation site for further copper growth, growing misoriented grains that embed the nanosheet within the film.

### 3.3 Corrosion Resistance

Figure S7 presents the corresponding OCP and Tafel curves for all the coated substrates including bare steel. In addition, Table S4 shows the parameters of the corrosion rates (*CR*), the corrosion inhibition efficiency (η), the polarization resistance (*R*_p_) as well the *OCP*, corrosion current density (*I*_corr_), and corrosion potential (*E*_corr_), which were derived from the analysis of Tafel curves. According to Figure S7a, the OCP values for the CMMC-coated samples (including Cu_Ref_) are shifted toward more positive potentials compared to the Ni-coated and bare steel samples. Similar behavior is recorded from the potentiodynamic polarization curves (Figure S7b) for the *E*_corr_. Such a shift implements the tendency of the coated samples to be more stable in the corrosive environment.^92^ In addition, all coated samples show a decrease in *I*_corr_ compared to bare substrate, with the largest decrease occurring for FGO-CMMCs. Due to this, FGO-CMMC exhibits the lowest corrosion rate of 1.9 μm yr^–1^, reaching the highest protection efficiency of 94.2%. Importantly, GO-CMMC also exhibits low CR, which is more than two times lower compared to Cu_Ref_ and Ni cases, indicating the anti-corrosion potential of CMMC coatings.

### 3.4 Tribological Testing at 1N Sliding Load

#### 3.4.1 Friction

**Figure 4:**
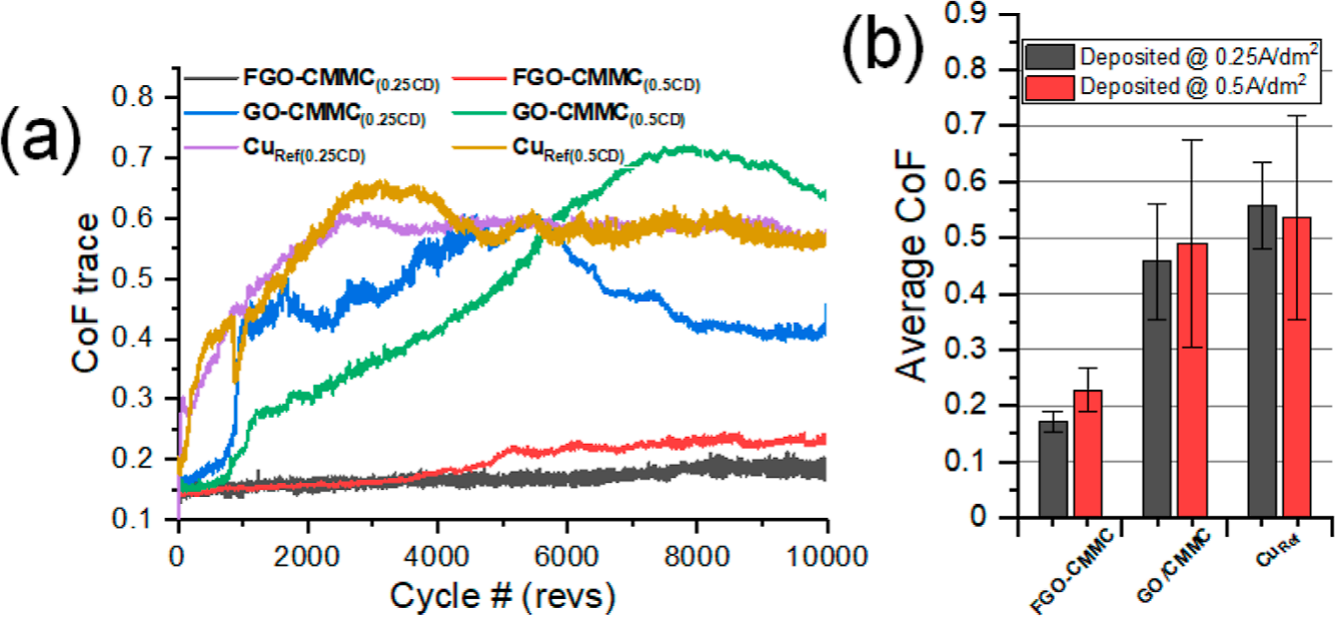
(a) Reciprocating PoD friction traces for FGO-CMMC, GO-CMMC, and Cu_Ref_ coatings at 1N sliding load. The bar chart in (b) is the average CoF calculated from the whole CoF trace for each sample run.

The coefficient of friction (CoF; μ) of the CMMCs was measured by reciprocating PoD sliding in ∼40% RH, under a normal load of 1 N (∼511 MPa maximum Hertzian contact pressure) selected to attempt to prolong the low-friction phase in the early phases of testing. Cu_Ref_ coatings typically show a rapid increase of friction after run-in, achieving μ > 0.3 in less than 500 revs and stabilizing at ∼0.6 until testing was stopped at 10,000 revs (Figure 4a). GO-CMMC gave tentative performances as a low-friction lubricating surface. Both GO-CMMC_(0.25CD)_ and GO-CMMC_(0.5CD)_ achieve a near-immediate low-friction sliding (μ ≤ 0.2) for a short period (approx. 750 revs), before lubrication fails and unstable, high-friction sliding modes are observed. In contrast, FGO-CMMC coatings show superior friction throughout the test period; a near-instant low-friction regime was observed at the start of FGO-CMMC_(0.25CD)_, sliding, and μ remained below 0.2 until tests were stopped at 10,000 revs of sliding (Figure 4b). FGO-CMMC_(0.5CD)_ displays similar low-friction running at the start of the sliding tests, but its performance fatigues after ∼4300 revs, followed by a slow rate of μ increase to ∼0.25 by the end of testing.

The sliding tests conclude that the incorporation of graphene oxide into a copper composite can generate a low-friction sliding surface. However, the longevity of the low-friction phase is owed to the function of the fluorinated groups in FGO.

#### 3.4.2 Wear

To reveal the lubrication and antiwear mechanisms of the CMMCs, the morphology of the wear track after sliding friction tests was analyzed by WL interferometry and SEM.

**Figure 5:**
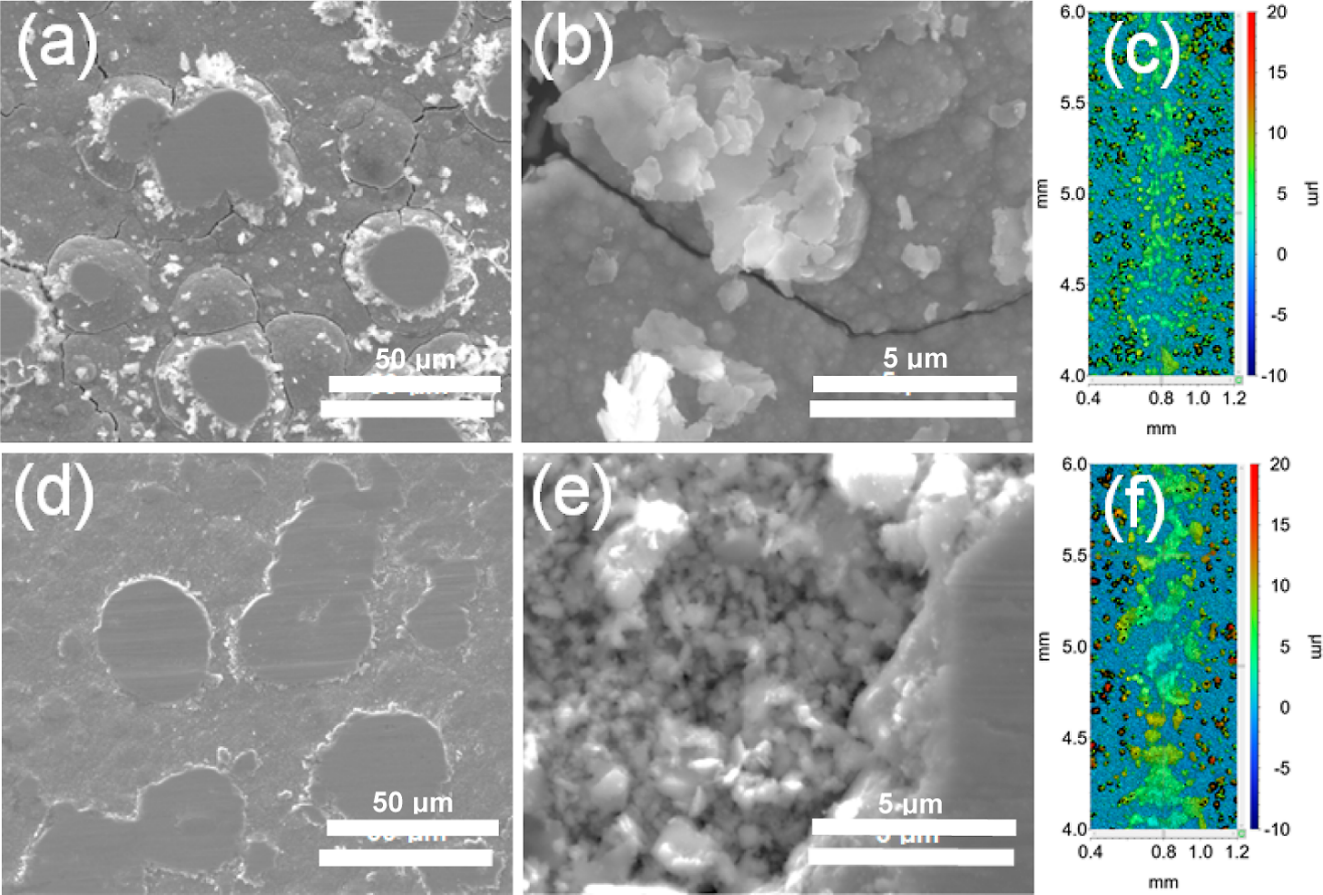
Imaging of the wear scars on FGO-CMMC coatings at 1N sliding load. SEM imaging of the wear on (a,b) FGO-CMMC_(0.25CD)_ and (d,e) FGO-CMMC_(0.5CD)_, identifying the wear tracks and debris generated from sliding testing. (c,f) WL interferometry topographs of the wear scars show no penetration into the bulk of the coating.

Optical and WL imaging on the sliding tracks on both FGO-CMMC_(0.25CD)_ and FGO-CMMC_(0.5CD)_ show that wear is limited to the top of the protruding misoriented growths, limiting the contact area on the coating; the high populous for these growths ensure that counterface-sliding does not contact the bulk copper coating (Figure 5). Observed wear depths after 10,000 revs sliding are limited to <5 μm, suggesting that the coatings can achieve a low-friction regime beyond the 10,000 revs endpoint selected. SEM imaging of the wear surface on FGO-CMMC_(0.25CD)_ showed clear evidence of shear-delamination: the contact surfaces are smooth, with the debris primarily consisting of platelet-structured materials. EDX mapping shows that the sliding contact surfaces are rich in carbon but contain no-enriched fluorine content. The debris surrounding the contact areas was also found to contain carbon and oxygen, suggesting that the ejected debris is from the oxidized FGO-Cu tribofilm. In contrast, FGO-CMMC_(0.5CD)_ showed a change in the lubrication performance at 4300 revs; the steady-state friction abruptly increased to CoF ∼0.25 and remained at this level until the end of the sliding test. This was accompanied by the observation of surface fatigue of the coating contact area as pitting and coating delamination were apparent by the ejection of larger microparticle debris and the increased erosion of the sliding surface. This is commensurate to small increases in interlayer shear energy, leading to increased oxidation at higher levels of friction.

The ∼9000 rev high-friction sliding observed during PoD testing of GO-CMMC_(0.25CD)_ and GO-CMMC_(0.5CD)_ is evident in post-test wear scar analysis as the misaligned growths on these coatings have a significantly higher degree of abrasive wear when compared to FGO-CMMC (Figure S8). Moreso, the concentration of misaligned crystal growth on the surface dictates the depth of wear observed. The GO-CMMC_(0.5CD)_ sliding track predominantly shows high wear of the embedded growths but limited penetration into the Cu-bulk coating. GO-CMMC_(0.25CD)_, on the other hand, shows penetration into the bulk-copper surface. GO-CMMCs succumb to wear of the bulk coating as a consequence of the lower area coverage of such growths on their surface (∼10% of the surface area, *c.f.* > 40% for FGO-CMMCs; Table S3). It is also noted that the high μ observed has given rise to coating fracturing, delamination, and erosion, resulting in microparticle-type debris redistributing along the sliding track. This was also observed after sliding on Cu_Ref_ coatings (Figure S8). The high-friction running on the coating is also anticipated to inhibit the formation of a robust tribofilm on the sliding track, as shown by the lack of surface carbon in the EDX map (Figure S9).

**Figure 6:**
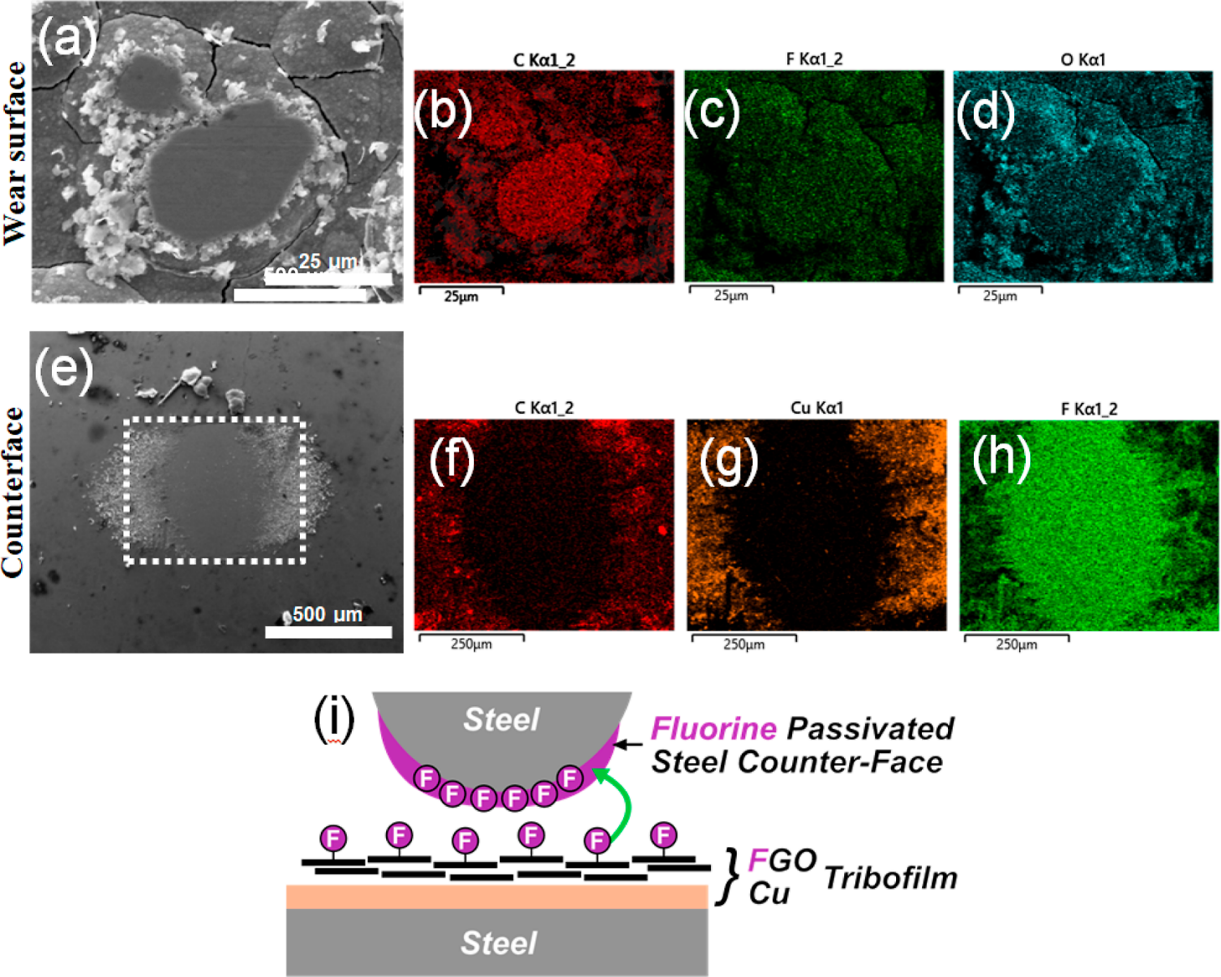
(a) SEM imaging and (b–d) EDX elemental mapping of a wear surface formed on FGO-CMMC_(0.25CD)_ after PoD sliding for 10,000 revs at 1N sliding load. (e) SEM image of the wear surface on the AISI 52100 counterface after PoD sliding on FGO-CMMC_(0.25CD)_, and (f–h) accompanied by elemental maps obtained by EDX analysis. (i) Schematic representation of the asymmetric tribofilm formation upon PoD sliding on FGO-CMMC coatings.

To further aid understanding, the morphology and elemental distributions of the AISI 52100 counterface ball from FGO-CMMC_(0.25CD)_ sliding were investigated by SEM and EDX (Figure 6). The SEM image of the counterface shows that the contact area has suffered minimal damage as the topography and roughness have not increased significantly [*c.f.* counterfaces from GO-CMMC_(0.25CD)_ and Cu_ref(0.25CD)_, sliding; Figure S8]. Raman analysis of the contact area shows no evidence of typical graphitic bands at 1360 and 1680 cm^–1^, indicating that no graphene-based transfer films are formed. Moreover, EDX maps of C, Cu, and F confirm that the counterface contains minimal copper and carbon on the surface, but its surface is fluorine-rich. The formation of the fluorinated steel surface is consistent with a tribochemical reaction between FGO and steel under a sliding load.

### 3.5 Tribological Testing of FGO-CMMCs at 2N Sliding Load

**Figure 7:**
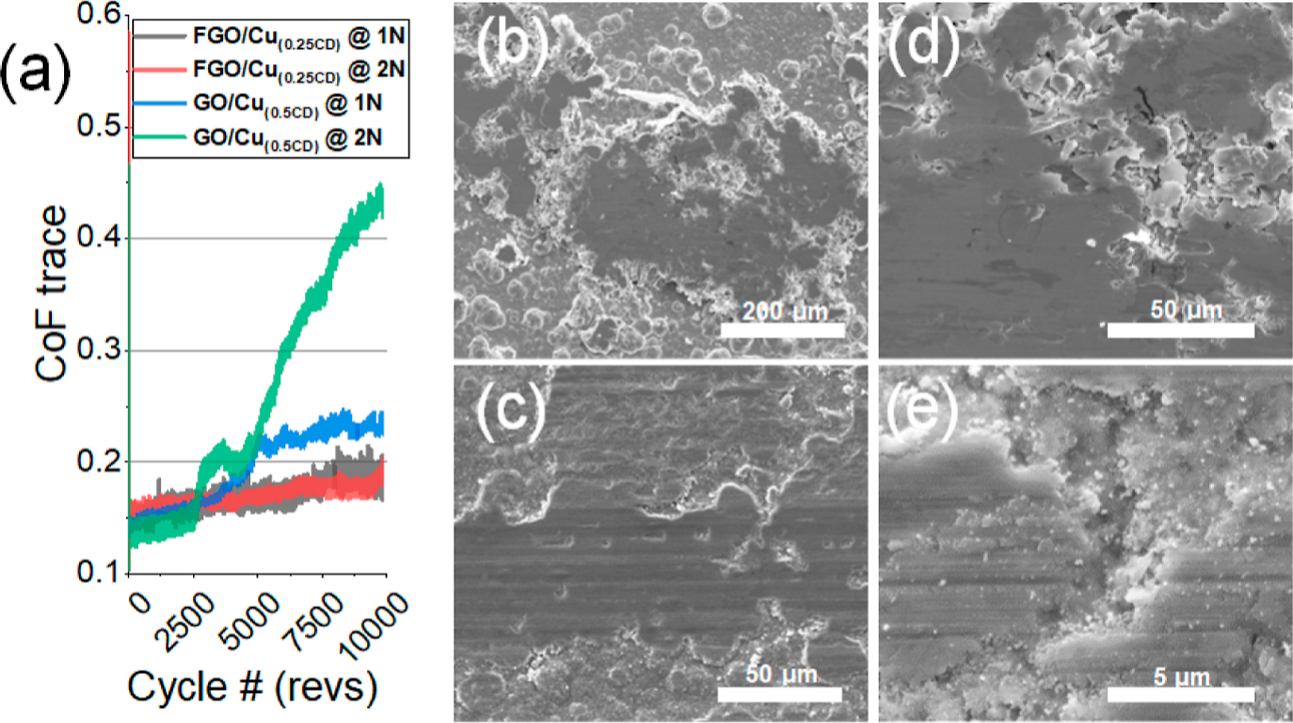
PoD results and SEM imaging of the wear scars on FGO-CMMC coatings at 2N sliding load. (a) CoF trace highlighting the superior performance of FGO-CMMC_(0.25CD)_ under 2N sliding load. SEM imaging of the wear on (b,c) FGO-CMMC_(0.25CD)_ and (d,e) FGO-CMMC_(0.5CD)_, identifying the wear tracks and debris generated from sliding testing.

To further identify the key lubrication modes within the system, FGO-CMMC_(0.25CD)_ and FGO-CMMC_(0.5CD)_ were also tested at 2N normal loads (∼644 MPa maximum Hertzian contact pressure). Similar to the results seen at the lower load, FGO-CMMC_(0.25CD)_ achieves a stable, low-friction performance throughout the test, maintaining a CoF <0.2 until the test ends (Figure 7a). In contrast, FGO-CMMC_(0.5CD)_ showed evidence of coating failure at 2N sliding loads; the fatiguing of the solid lubricant initiates at ∼ 2700 revs, initially increasing the steady-state CoF to ∼0.23, before gradual failure is observed after 4000 revs, with μ increasing to >0.4 by the end of the 10,000 revs test.

SEM imaging of the wear surfaces on the FGO-CMMC_(0.25CD)_ track at the higher load shows increased wear of the misoriented growths; however, the shear-delamination is still dominant: the debris within the tracks is sheared-platelet like (Figure 7b,c). This is in contrast to FGO-CMMC_(0.5CD)_ as the micrographs show high rates of wear and microparticle-like debris generation (Figure 7d,e). This supports the theory that the more ductile mechanical properties of FGO-CMMC_(0.25CD)_ result in a sliding surface that is more resistant to abrasive wear, promoting a longer-lasting lubricating tribofilm.

### 3.6 Lubrication Mechanism of FGO-CMMC and GO-CMMC Coatings

The macroscale low-friction performance of FGO-CMMCs and GO-CMMCs is driven by the sliding interface between the graphene-copper tribofilm and the steel counterface, but low-friction lifetimes and antiwear performances are influenced by the formation of a robust fluorine-rich transfer layer on the counterface. Graphene is known to not easily form a tribolayer onto steel surfaces due to its chemical inertness.^93^ Conversely, the oxygen-functional groups on graphene oxide are susceptible to adhesion onto metal surfaces,^94^ as well as inhibiting low-shear interactions between nanosheets, therefore, accelerating damage of the nanosheets under sliding. Liu et al.^42^ recently demonstrated that sliding fluorographene oxide coatings on steel generates a metal-fluoride surface on the steel counterface, promoting a robust transfer film^95^ that facilitates a low-shear sliding mechanism between steel contact pairs. However, due to the nature of the contact pair in FGO-CMMC sliding, there is no evidence of carbon on the transfer film, suggesting that the tribofilm structure is asymmetric: the coating reconstructs under a sliding load to a lamellar FGO/Cu tribofilm, sliding against the fluorine-passivated steel counterface, it is this structure that provides the long-lasting, low-friction performance (Figure 6i). This is beneficial for low-friction longevity as the fluorine-rich transfer film is continually regenerated by the gradual wear and reconstruction of the FGO/Cu tribofilm, further retarding coating failure. The robustness of FGO-CMMC_(0.25CD)_ at higher loads is attributed to the improved ductility of the composite (c.f. pure copper) that further reduces the coatings’ macroscale interlaminar shear strengths, allowing for a more efficient construction and maintenance of the tribofilm during sliding.

The FGO within the CMMC forms a robust tribofilm on steels that exhibits excellent low-friction and antiwear properties. Therefore, the FGO-CMMC is a promising solid lubricant for mechanical engineering applications.

## 4 Conclusions

Low-profile FGO-CMMC coatings were uniformly deposited onto AISI 52100 bearing steels by electrodeposition and exhibited excellent lubrication performances under 1N macroscale sliding loads, reducing the average coefficient of friction by 69 and 63% over an extended test period, compared to coatings of pure copper and GO-CMMC with similar topography. In addition, FGO-CMMC exhibits the highest corrosion protection efficiency among the examining samples, reaching 94.2% compared to the bare substrate, enhancing the low-friction performance. The improved lubrication performance and wear resistance of the FGO-containing CMMCs than those with GO were attributed to the construction of a robust, asymmetrically structured sliding tribofilm that promotes low-friction sliding and reduced wear. The improved ductility of FGO-CMMC_(0.25CD)_ allows for further reductions in interlaminar shear strengths of the coating, contributing to robust performances at higher sliding loads. The ease of FGO-CMMC composite deposition, combined with its excellent lubrication performance, improved anti-corrosion properties, and wear resistance makes it a promising solid lubricant for its use as a lubricating medium within steel-based mechanical switch assemblies.

## Supporting Information Available

Full characterization of the GO and FGO powders, chemical characterization of the GO_(filtrate)_ and FGO_(filtrate)_ extracted nanosheet characterization of the as-prepared GO-CMMC and Cu_Ref_ coatings, and post-wear analyses on GO-CMMC and Cu_ref_ coatings (PDF)
