# Supplementary material for: Tribology of Copper Metal Matrix Composites Reinforced with Fluorinated Graphene Oxide Nanosheets: Implications for Solid Lubricants in Mechanical Switches
Source: ACS Appl Nano Mater. 2023 May 10;6(10):8202–13. doi: 10.1021/acsanm.3c00399 (PMC10227772; doi:10.1021/acsanm.3c00399)
Supplement: Supplementary file 1 — an3c00399_si_001.pdf [file an3c00399_si_001.pdf]

Supporting Information

Tribology of Copper Metal-Matrix Composites Reinforced with Fluorinated Graphene Oxide Nanosheets: Implications for Solid Lubricants in Mechanical Switches

Nicky Savjani[a], Vicente Orts Mercadillo[a], Darren Hodgeman[b], George Paterakis[c], Yubao Deng[a], Cristina Vallés[a], George Anagnostopoulos[c], Costas Galotis[c,d], Mark A. Bissett[a] and Ian A. Kinloch[a]**

[a] Department of Materials, Henry Royce Institute and National Graphene Institute, The University of Manchester, Oxford Road, M13 9PL, UK.

[b] Carbon Science Center of Excellence, Morgan Advanced Materials and Technology, Inc., 310 Innovation Boulevard, Technology Center, Suite 250, University Park, PA 16803, USA.

[c] Foundation for Research and Technology Hellas, Institute for Chemical Engineering Sciences, Stadiou St, Platani, Patras, GR26504, Greece.

[d] Department of Chemical Engineering, University of Patras, Patras 26504, Greece

E-mails: mark.bisset@manchester.ac.uk and ian.kinloch@manchester.ac.uk

GO and FGO powder characterisation

Characterization of the GO and FGO powders was carried out using a variety of techniques. Transmission electron microscopy (TEM) was performed using a Tecnai T20 scope. Samples were prepared by drop casting dispersions of 0.1 mg/ml (F)GO in 1:1 water, IPA onto a lacey carbon support. SEM was carried out using a Zeiss Ultra 55 FEG scope. Samples were prepared by drop casting dispersion of 1 mg/ml (F)GO in 1:1 water, IPA onto a silicon wafer support. Contact angle measurements were taken on a Biolin Scientific, Theta Lite optical tensiometer. A 2 mL water droplet was pipetted onto the pelletized powder (13mm discs, consisting of 0.5g of powder, pressed at 10 tons for 5 minutes using a hydraulic press). Laser diffraction particle sizing was carried out using a Malvern Mastersizer, with the HydroEV attachment. Samples were dispersed in 1:1 water, IPA solution until appropriate obscuration values were reached and measured post two rounds of 120s sonication. Bulk FTIR spectra were obtained from 32 co-averages collected in transmission mode using an ATR-FTIR spectrometer (Nicolet iS50 spectrometer, Thermo Scientific) with a diamond crystal window, operating at 1 cm^{-1} resolution across a 650-4000 cm^{-1} range. Raman Spectroscopy was carried out on a Renishaw InVia with a 532 nm laser and a 50x scope. The StreamHR mapping function was used to map 480 spectra over a ~2000 μm^2 area.

X-ray Photoelectron Spectroscopy (XPS) was performed on an Axis Ultra Hybrid spectrometer (Kratos Analytical) using monochromated Al Kα radiation (1486.6 eV, 10 mA emission at 150 W, spot size 300 x 700 μm) with a base vacuum pressure of $\sim 5 \times 10^{-9}$ mbar. Charge neutralization was achieved using a filament. Analysis was carried out in CasaXPS.[1] Binding energy scale calibration was performed using C-C in the C 1s photoelectron peak at 284.7 eV. The high-resolution (HR) C_{1s} peaks were fitted using a finite Lorentzian (LF) peak shape, with a damping parameter used to quash the peak tail.[2] A three-parameter Tougaard background (U 3) was used and other component peaks were modelled as symmetric Voigt line shapes LA(1,600), with a consistent FWHM. The number of component peaks was initially approximated by eye and refined in subsequent fittings to achieve a random residual distribution. A Shirley background and symmetric Voigt components of equal FWHM were used to deconvolute HR-O_{1s}. A three-parameter Tougaard background and symmetric Voigt components of equal FWHM were used to deconvolute HR-F_{1s}. The transitions with the largest peak areas were selected for quantification. In the case of Cu_{2p}, K_{2p} and P_{2p}, the doublet pairs were used and scaled using the total relative

sensitivity factor (RSF).[1] The K_{2p} doublet overlaps with the C_{1s} peak, as such a synthetic peak model was fitted to apportion the area.[1] Since K_{2p} spin-orbit splitting gives rise to a doublet, the component areas of 1/2 and 3/2 are constrained to a ratio of 0.5.

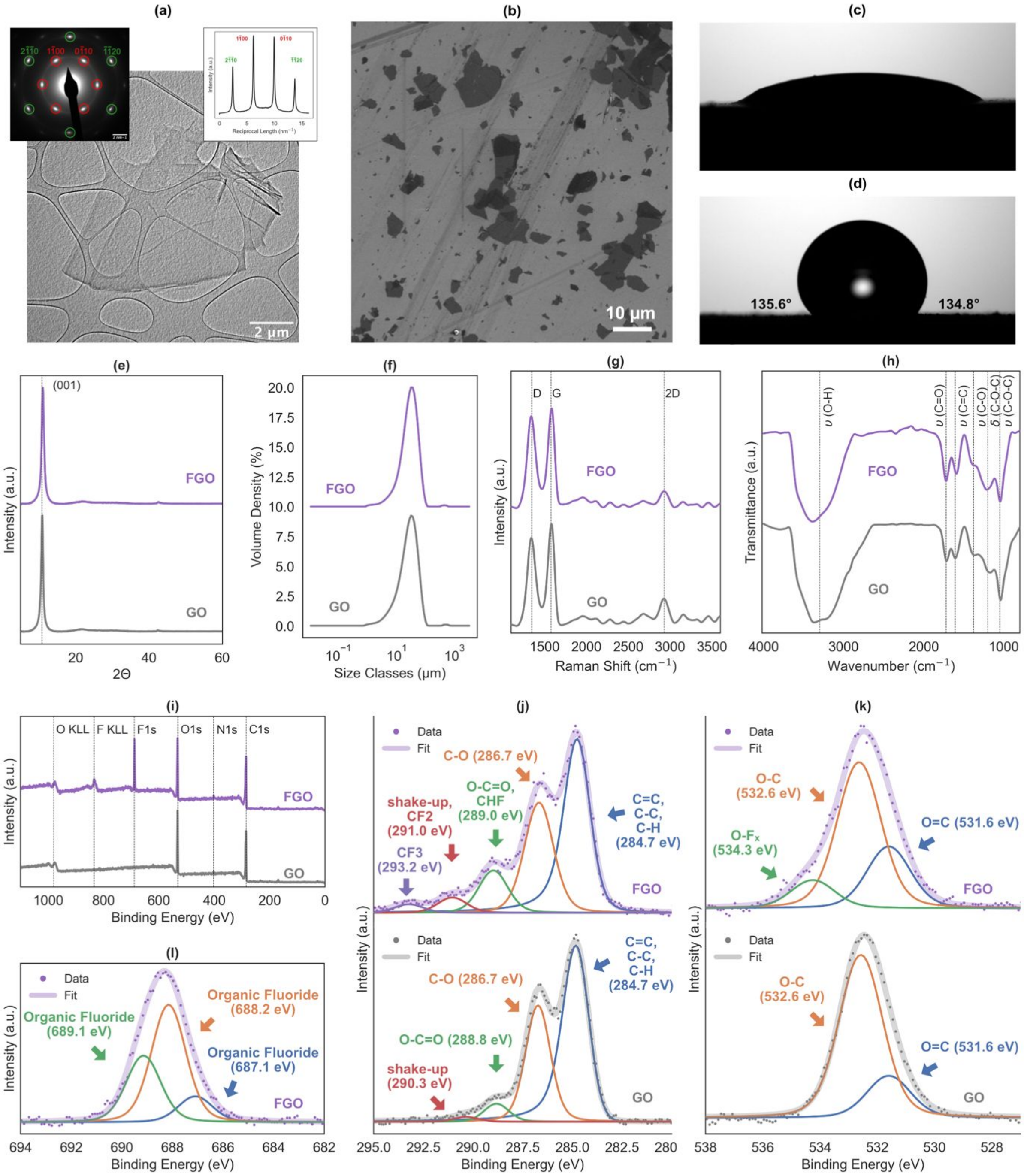

Figure S1. (a) TEM image of a GO flake on a lacey carbon support and its selected area electron diffraction pattern (inset top left) and accompanying intensity profile (inset top right). SEM of an image of GO sheets drop-cast onto a silicon wafer. (c, d) GO and FGO contact angle measurement stills. (e-h) Characterisation of GO and FGO using XRD, laser-diffraction particle sizing, Raman spectroscopy, and FTIR. (i) XPS Survey scan for GO and FGO. (j-l) High-resolution C_{1s}, O_{1s} and F_{1s} XPS scans for GO and FGO.

The detailed characterization of FGO and GO powders is summarized in Figure S1. A TEM image of a typical GO flake is shown in (a), its transparent nature indicating few-layer material, the inset (top left) selected area electron diffraction pattern (SAED), taken at the centre of the flake shows a hexagonal pattern of spots (annotated with Miller-Bravais *hkil* notation) extending across the captured image. The linear intensity profile of the SAED pattern in the inset (top right), shows that the intensity ratio between 1100 and 2110 is 1.7, this is consistent with few-layer behaviour[3] and follows literature values for GO.[4] No diffraction spots from the oxygen moieties are seen, as expected since these do not produce the requisite ordered lattice arrays.[4]

The distribution of lateral size can be visualized in the SEM image of GO flakes drop cast onto a silicon wafer in (b). The consistent contrast indicates flakes of similar thickness, while a range of flake sizes can be observed from < 1 μm, to > 10 μm. The particle size distribution in (f) shows an asymmetric distribution for GO and FGO, as the blended freeze-dried powder had been sieved to < 50 μm, to minimize the presence of agglomerates. The D_{50} is 28.5 and 30.5 μm for GO and FGO respectively. The slight increase could be indicated by larger aggregates in the FGO, possibly due to worse dispersion in the water and IPA solution due to the presence of hydrophobic fluorine groups. The characteristic (001) peak can be observed for both GO (at 10.9°) and FGO (at 11.0°) in the XRD spectra in (e), from which the interlayer spacing is calculated to be 8.1 and 8.0 Å respectively, suggesting that there is minimal effect on sheet-stacking post-fluorination.

The effect of fluorination can be most clearly observed in contact angle measurements, as shown in (c, d) for GO and FGO respectively. For the former, the hydrophilic surface leads the water to spread across the field of view, and so the contact angle is too small to accurately measure. Whereas for the latter, significant hydrophobic behaviour is observed due to the presence of fluorine groups increasing the contact angle to 135.2°.

The average FGO and GO spectra for the mapped area are shown in (g). Each spectrum shows a characteristic graphitic G mode at ~1566 cm^{-1} and defect-induced D mode at ~1342 cm^{-1} and weak 2D mode at ~2930 cm^{-1}. The I_D/I_G ratio increases from 0.93 (±0.02) for GO to 0.97 (±0.05) for FGO. However, since the FWHM of the G peaks is above 30 (61.2 and 56.8 cm^{-1} for GO and FGO respectively), the material is in the second stage of defect evolution, wherein an increase in I_D/I_G indicates a reduction in disorder.[5] Indicating that the plasma

fluorination acts to clean the graphitic surface of amorphous debris and/or passivate defects. The (F)GO powders were analysed under ATR-FTIR, as shown in (h). Both materials share a similar spectrum, hydroxyl (-OH) groups display a broad peak of ~3400 cm^{-1}, while other carbon lattice and oxygen functionality-derived peaks appear between 2000 – 1000 cm^{-1}. These consist of carboxylic stretching from C=O at 1720 cm^{-1} and C-OH at 1380 cm^{-1}[6], aromatic C=C stretching at 1610 cm^{-1} [7], and epoxy (C-O-C) deformations and stretching at 1200 and 1050 cm^{-1} respectively[8, 9]. Fluorination appears to slightly dampen both carboxylic and epoxy peaks, implying functionalisation occurs via the substitution of these oxygen moieties with fluorine.

Table S1: Relative atomic composition (at.%) of (F)GO powders.

Material	C_{1s}	O_{1s}	N_{1s}	F_{1s}
GO	76.6 (±0.4)	22.6 (±0.8)	0.8 (±1.2)	0.0
FGO	68.4 (±1.0)	20.2 (±0.5)	0.7 (±1.1)	10.8 (±0.9)

The XPS survey scans for the (F)GO powders in (i) show intense C_{1s}, O_{1s} and in the case of FGO, F_{1s} peaks, as well as a weak N_{1s} peak in both spectra. The relative atomic composition of each is summarised in Table S1. The increase in fluorine content comes primarily at the expense of 8.2 at.% carbon and 2.4 at.% oxygen suggesting that the fluorine is primarily passivating existing carbon defects or creating new ones, and only weakly displacing existing oxygen moieties.

The HR-C_{1s} (F)GO scans in (j) show a broad and asymmetric peak, due to the highly defective graphitic nature of the materials. This can be deconvoluted into a neutral carbon peak (C-C, C=C, C-H) at 284.7eV. For GO, three additional components were observed: C-O (286.7 eV), O-C=O (288.8 eV) and a shake-up feature (290.3 eV).[2] Assigning peaks in FGO powder is more complex as the fluorine chemical states overlap with existing oxygen-derived functionalities. CHF overlaps with the O-C=O peak at 289.0 eV, CF_2 overlaps with the shake-up at 291.0 eV, both of which broaden and increase in intensity, and a new CF_3 peak appears at 293.2 eV.[10] The HR-O_{1s} scans in (k) show two components for GO, a C=O peak (531.6 eV) and a C-O peak (532.6 eV).[11] These are present in FGO, alongside the O-F_x peak (534.3 eV).[12] Indicating, that fluorination occurs both through oxygen modalities and the carbon skeleton. The HR-F_{1s} for FGO in (l) can be deconvoluted into three component peaks centred between 687 – 689 eV assigned to organic fluorides.

GO and FGO copper-plating dispersion filtrate characterisation

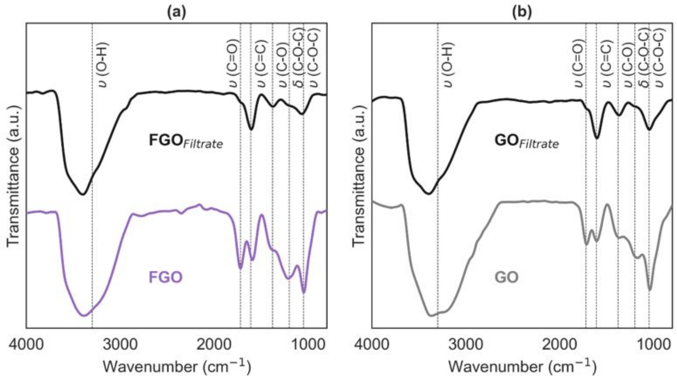

Figure S2: (a) FGO and (b) GO powder and corresponding copper plating dispersion filtrate.

Following the discovery of heterogeneous growth processes and increased nanosheet interlayer spacing in the **FGO-CMMC** and **GO-CMMC** coatings, the nature of the 2D materials within **(F)GO-Copper** plating dispersions was investigated further. Enabling a better understanding of the chemical modifications imposed on the (F)GO powders by combining them with the copper plating solutions. To do this, 2 mL aliquots of the 1 mg/mL **(F)GO-Copper** plating dispersions were extracted and diluted with 8 mL deionised water. The diluted solutions were passed through a PVDF filter via a syringe filtration assembly (13 mm diameter, 0.1 μm pore size, syringed at 10 mL/h), followed by two 10 mL DI water washes. The resulting filtrates (called $\mathbf{FGO_{Filtrate}}$ and $\mathbf{GO_{Filtrate}}$) were dried *via vacuo* for 4 hours at 40 °C before characterisation. The results shown are for material plated for 6 hours at 0.25 A/dm^2 and are representative of both un-plated filtrates and filtrates plated for 3 hours at 0.5 A/dm^2.

The ATR-FTIR spectra of the $\mathbf{(F)GO_{Filtrate}}$, have been plotted alongside the relevant powder in Figure S2 to better discern spectral changes. In both cases, the hydroxyl and aromatic C=C peaks are unaffected, however, the intensity of the other oxygen functionality peaks is dampened, in particular the carboxyl C=O (1720 cm^{-1}) peak. The lack of bands in the alkyl C-H region (2900-2750 cm^{-1}) eliminates organic functionalisation, suggesting this is due to carboxyl-edge functionalisation by an inorganic component.

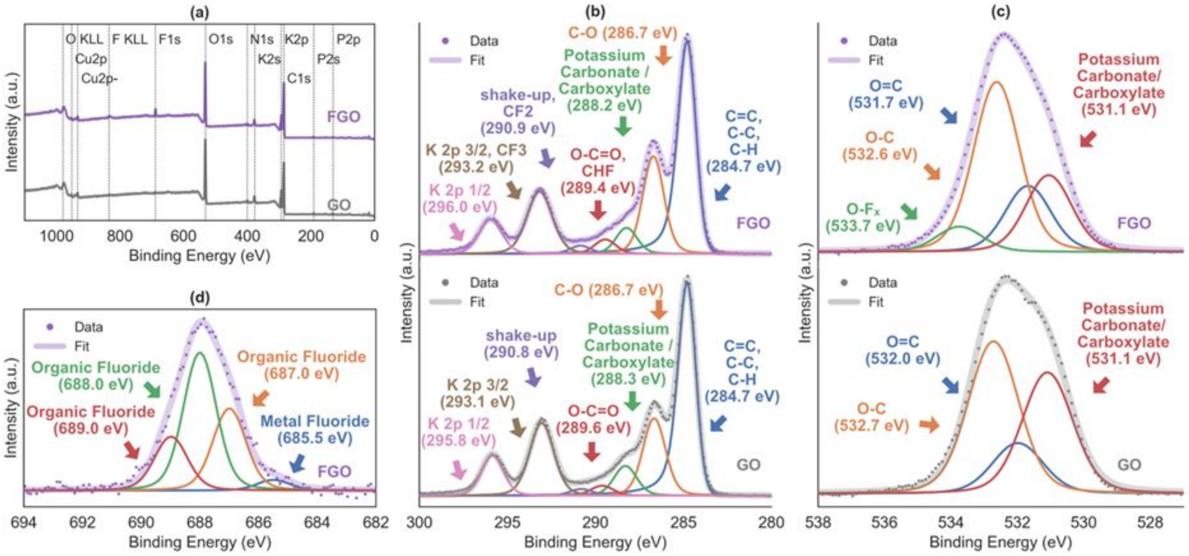

Figure S3: (a) XPS survey scan and (b-d) high-resolution C_{1s}, O_{1s} and F_{1s} scans for (F)$GO_{Filtrate}$ samples.

The XPS analysis for the **(F)$GO_{Filtrate}$** samples is summarised in Figure S3. The survey scans in (a) show the change in elemental composition post-dispersion: The presence of copper, potassium and phosphorous peaks is expected as these are contained in the bath mixture. The relative atomic composition of each is summarised in Table S2. The potassium is present in larger quantities (4.9 and 4.2 at. % for **$GO_{Filtrate}$** and **$FGO_{Filtrate}$**) than copper or phosphorus, indicative of favoured potassium-functionalisation on the (F)GO nanosheets. The drop in relative fluorine content in the FGO post-dispersion (-8.7 at.%) suggests that fluorine groups are being displaced.

Table S2: Relative atomic composition (at.%) of (F)GO copper-plating dispersion filtrate.

Material	C_{1s}	O_{1s}	N_{1s}	F_{1s}	K_{2p}	Cu_{2p}	P_{2p}
**$GO_{Filtrate}$**	68.2 (±0.2)	24.7 (±0.1)	1.0 (±0.1)	0.0	4.9 (±0.1)	0.8 (±0.04)	0.4 (±0.1)
**$FGO_{Filtrate}$**	69.1 (±0.5)	23.5 (±0.2)	0.7 (±0.1)	1.9 (±0.4)	4.2 (±0.03)	0.5 (±0.05)	0.2 (±0.07)

The HR-C_{1s} peak for the **(F)$GO_{Filtrate}$** samples overlaps with K_{2p} peaks so both have been fitted simultaneously in (b). A new peak appears at ~288.2 eV assigned which can be assigned to C_{1s} signals for potassium carbonate/carboxylate, further evidencing functionalisation with the potassium metal in dispersion.[13] The shake-up, CF_2 and O-C=O,

CHF peaks have a higher relative area in the FGO scan, which could be attributed to any fluorine that remains post-deposition. The HR-O_{1s} peak for the **(F)GO$_{Filtrate}$** samples, shown in (c), broadens towards lower binding energies, to encompass a potassium carbonate/carboxylate peak (~ 531 eV). The O-F_x peak weakens in the FGO post-deposition, in line with the drop in atomic fluorine concentration. In the HR-F_{1s} peak for **FGO$_{Filtrate}$**, shown in (d), a new peak appears at 685.5 eV, indicating the presence of metallic fluoride bonds.[14] This suggests that the displaced fluorine groups may go on to react with potassium ions in solution and filter out as salts on the **FGO$_{Filtrate}$**.

GO-CMMC and Cu$_{Ref}$ coating characterisation

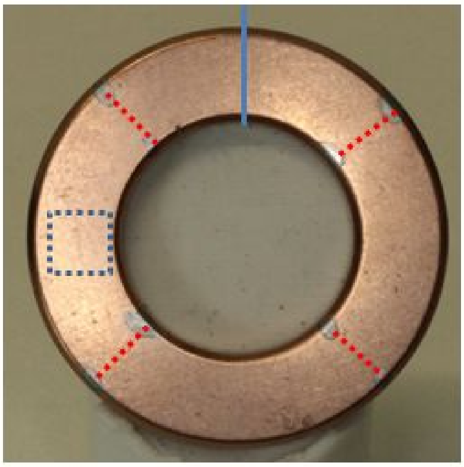

Figure S4. Photograph of a representative CMMC. The samples were orientated in the same manner before measurement (the point where each sample was hung is indicated by the blue solid line). Before deposition, latex masks were applied at 8 locations to inhibit deposition for coating thickness determination. The thicknesses were measured across the four lines in red and averaged. The blue box denotes the 9mm^2 section where the roughness was analysed in each sample by white light interferometry.

Cu$_{Ref}$ coatings typically showed good uniformity (Figure S6c, d, g, h, k, and l) and thicknesses close to the ascribed 20 μm target (Figure S5). White light interferometry showed that **Cu$_{Ref(0.25CD)}$** and **Cu$_{Ref(0.5CD)}$** coatings are deposited uniformity across the whole substrate with R_a values of between 0.2 and 0.3 μm, consistent with the roughness of the underlying substrate (~0.25 μm).

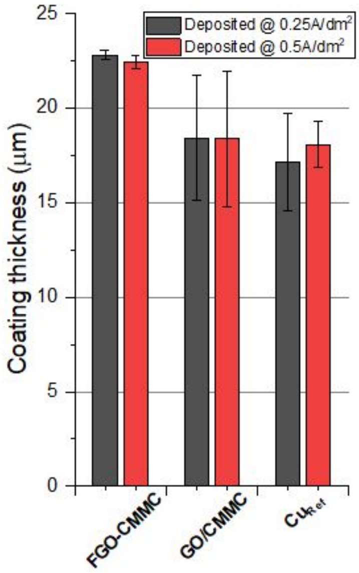

Figure S5. The measured thicknesses of FGO-CMMC, GO-CMMC and Cu$_{Ref}$ coatings. The values are based on the average thickness measured across each of the paired masked areas.

GO-CMMC coatings show a similar coating structure as **FGO-CMMCs**; a slight visual roughening of the surface with evidence of misaligned growths on the surface. SEM and WL interferometry imaging shows that the density of growths in GO-CMMCs is significantly lower, but comprises larger grains (Figure S6 a, b, e, f). SEM imaging of **GO-CMMCs** (Figure S6i, j) coatings reveals that GO nanosheets are embedded and protruding from the core of the misorientated grains, with no copper nanoparticle growth on its surface,

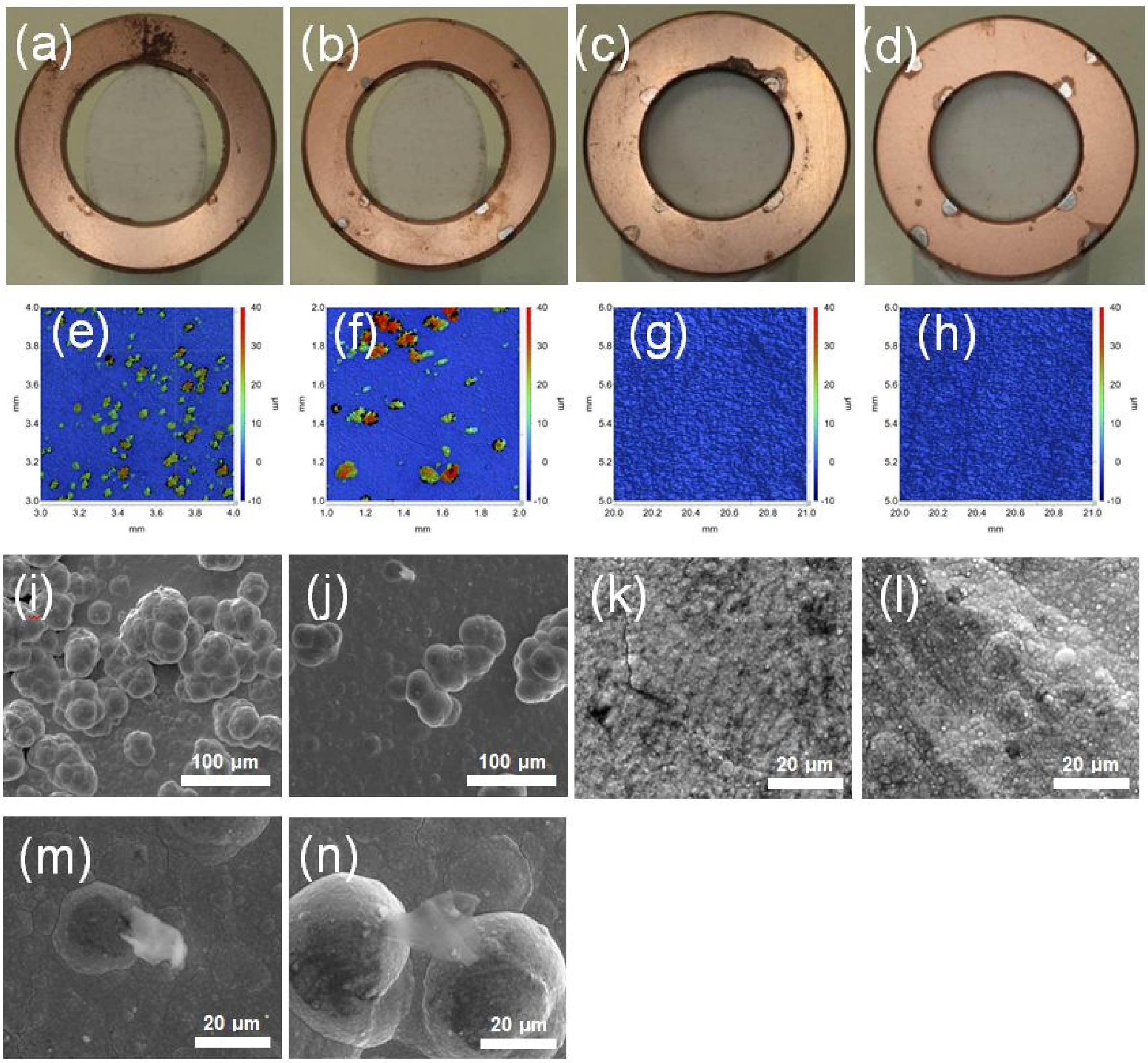

Figure S6. Camera (a-d), White-light interferometry (e-f) and electron microscopy (g-n) images of the GO-CMMC and Cu_{Ref} coatings electrodeposited onto AISI 52100 steel: (a, e, i and m) GO-$CMMC_{(0.25CD)}$ (b, f, j and n) GO-$CMMC_{(0.5CD)}$, (c, g and k) $Cu_{Ref(0.25CD)}$, (d, h and l) $Cu_{Ref(0.5CD)}$.

Table S3. Statistical analysis from the topographical data of the FGO-CMMC and GO-CMMC coatings. All measurements were carried out from a 3.75 mm^2 area of each coating.

Coating	Number of growths	Coverage of growths (% area)	Average growth height (μm)	Avg. surface area per growth (mm^2)	Roughness (μm)
**FGO-CMMC$_{(0.25CD)}$**	2007	51.0	12.8 (± 4.4)	0.0036 (± 0.006)	2.8
**FGO-CMMC$_{(0.5CD)}$**	2005	41.0	17.5 (± 5.9)	0.0029 (± 0.003)	3.1
**GO-CMMC$_{(0.25CD)}$**	299	9.9	36.0 (± 9.2)	0.0046 (± 0.005)	2.6
**GO-CMMC$_{(0.5CD)}$**	297	10.9	32.8 (± 9.2)	0.0051 (± 0.006)	1.2

Corrosion protection studies on FGO-CMMC, GO-CMMC and Cu_{Ref} coatings

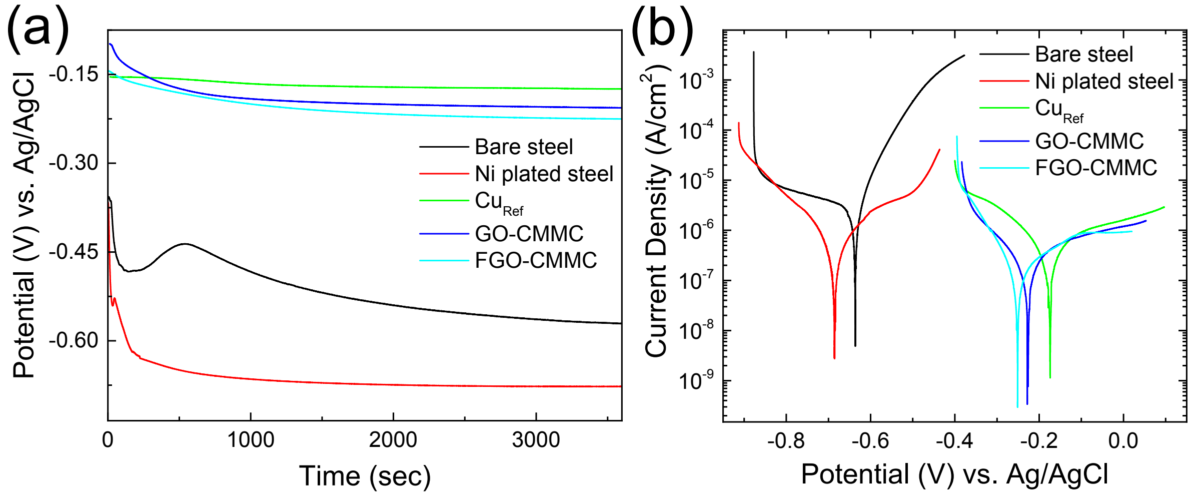

Figure S7. OCP (a) and Tafel (b) curves obtained using NaCl 3.5 % as corrosion electrolyte for bare steel, Ni-plated steel, Cu_{Ref}, GO-CMMC and FGO-CMMC

The corrosion rate (CR) calculation is based on Faraday's Law, according to the following expression[15]:

$$CR = K \cdot EW \frac{I_{corr}}{\rho} \quad \text{(Eq. 1)}$$

where K is the corrosion constant 3272 mm Kg A^{-1} cm^{-1} yr^{-1}), EW is the equivalent weight and ρ the density of the steel, respectively.

In addition, for the evaluation of the corrosion inhibition efficiency η, the following expression is used[16]:

$$\eta = \frac{I_{corr,\,sub} - I_{corr,\,coat}}{I_{corr,\,sub}} \quad \text{(Eq. 2)}$$

While the polarization resistance R_p is estimated based on the Stern-Geary equation[17], using the anodic (b_a) and cathodic (b_c) slopes of the Tafel plots:

$$R_p = \frac{b_a b_c}{2{,}303 \cdot I_{corr}(b_a + b_c)} \quad \text{(Eq. 3)}$$

A summary of all the results presented in Figure S7a and b, are given in Table S4 below.

Table S4. Calculated corrosion parameters for all the samples measured in NaCl 3.5 %

Coating	OCP (mV)	E_{corr} (mV)	I_{corr} (μA cm^{-2})(	b_c (V dec^{-1})	b_a (V dec^{-1})	CR (μm yr^{-1})	R_p (Ω cm^{-2})	η (%)
**Bare steel**	-571.1	-636.1	2.81	435.9	57.4	32.7	7.8	
**Ni coted steel**	-677.1	-685.9	0.54	118.9	136.4	6.3	50.8	80.7
**Cu_{REF}**	-174.3	-174	0.48	156.8	265.5	5.5	90.0	83.1
**GO-CMMC**	-206.6	-228.2	0.23	131.2	206.9	2.6	153.6	91.9
**FGO-CMMC**	-225.2	-251.2	0.16	78.8	196.5	1.9	150.8	94.2

Post-wear analysis on GO-CMMC and Cu_{Ref} coatings

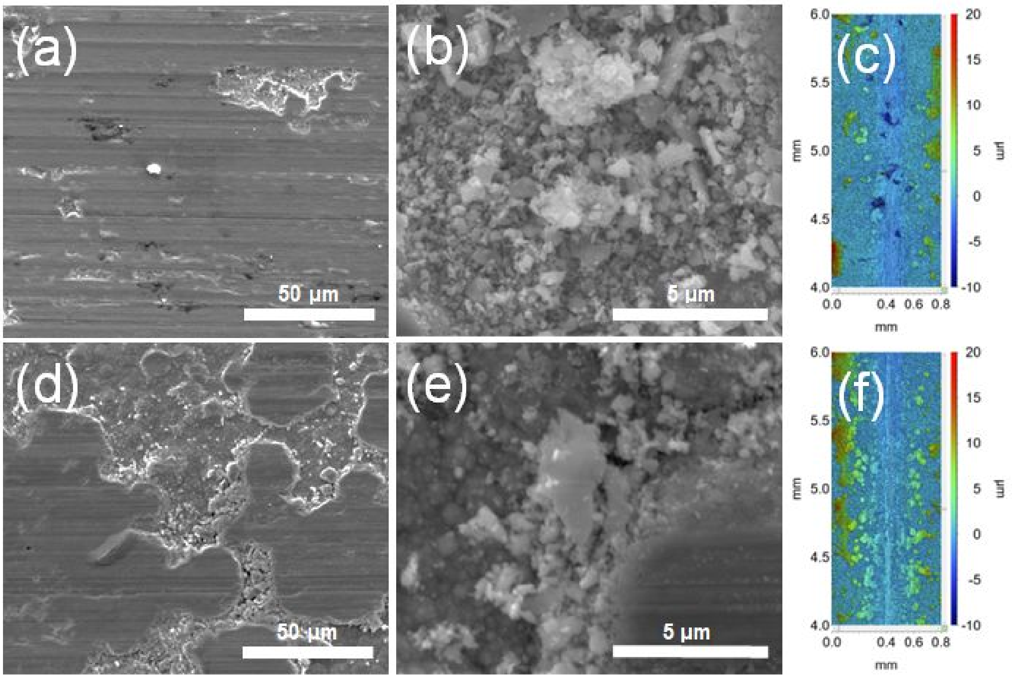

Figure S8. SEM and WL interferometry imaging of the wear scars on the sliding tracks of GO-CMMC coatings. The wear scars on (a-c) GO-CMMC$_{(0.25CD)}$ and (d-f) GO-CMMC$_{(0.5CD)}$.

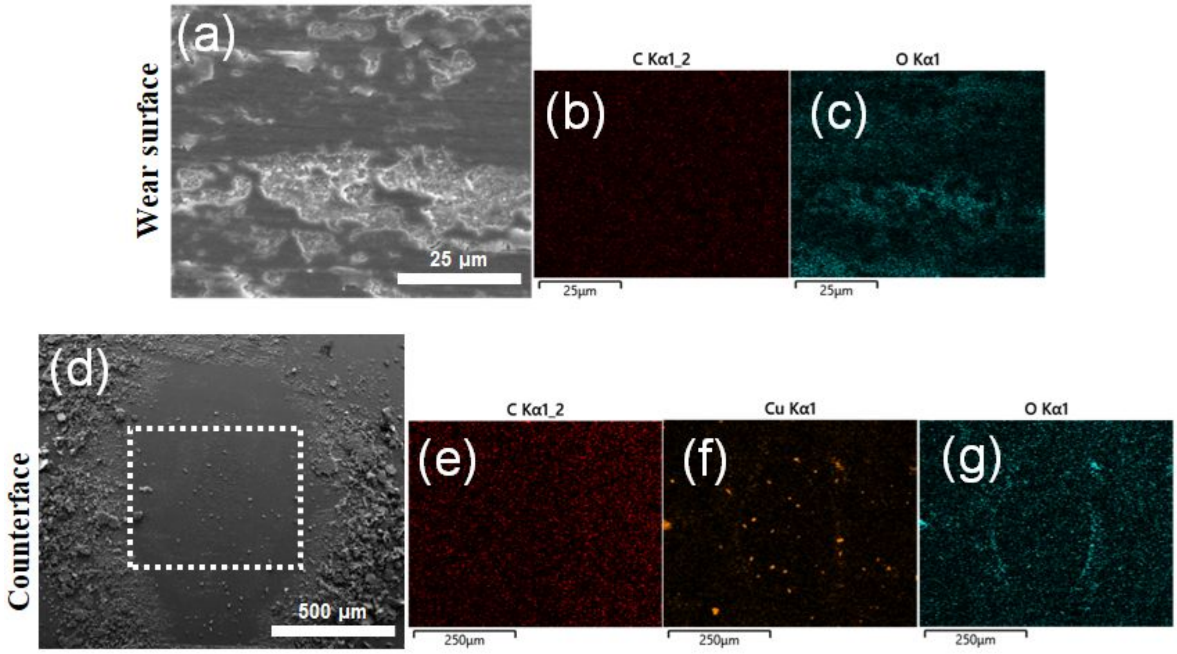

Figure S9. (a) SEM imaging and (b, c) EDX mapping of a wear surface formed on GO-CMMC(0.25CD) after PoD sliding for 10,000 revs. (d) An SEM image of the wear surface on the AISI 52100 counterface ball after PoD sliding on GO-CMMC$_{(0.25CD)}$, (e-g) accompanied by elemental maps obtained by EDX analysis

References

1. Walton, J.; Wincott, P.; Fairley, N.; Carrick, A., *Peak Fitting with CasaXPS*. Accolyte Science: Knutsford, UK, 2010.

2. Gengenbach, T. R.; Major, G. H.; Linford, M. R.; Easton, C. D., Practical Guides for X-Ray Photoelectron Spectroscopy (XPS): Interpreting the Carbon 1s Spectrum. *J. Vac. Sci. Techn. Vac. Surf. Films* **2021,** *39* (1), 013204. DOI: 10.1116/6.0000682.

3. Meyer, J. C., *et al.*, On the Roughness of Single- and Bi-Layer Graphene Membranes. *Solid State Commun.* **2007,** *143* (1-2), 101. DOI: 10.1016/j.ssc.2007.02.047.

4. Wilson, N. R., *et al.*, Graphene Oxide: Structural Analysis and Application as a Highly Transparent Support for Electron Microscopy. *Acs Nano* **2009,** *3* (9), 2547. DOI: 10.1021/nn900694t.

5. Nagyte, V., *et al.*, Raman Fingerprints of Graphene Produced by Anodic Electrochemical Exfoliation. *Nano Lett.* **2020,** *20* (5), 3411. DOI: 10.1021/acs.nanolett.0c00332.

6. Lee, D. W., *et al.*, The Structure of Graphite Oxide: Investigation of Its Surface Chemical Groups. *J. Phys. Chem. B* **2010,** *114* (17), 5723. DOI: 10.1021/jp1002275.

7. Ţucureanu, V.; Matei, A.; Avram, A. M., FTIR Spectroscopy for Carbon Family Study. *Crit. Rev. Anal. Chem.* **2016,** *46* (6), 1. DOI: 10.1080/10408347.2016.1157013.

8. Johra, F. T.; Jung, W.-G., Hydrothermally Reduced Graphene Oxide as a Supercapacitor. *Appl. Surf. Sci.* **2015,** *357*, 1911. DOI: 10.1016/j.apsusc.2015.09.128.

9. Díez-Pascual, A. M.; Díez-Vicente, A. L., Poly(propylene fumarate)/Polyethylene Glycol-Modified Graphene Oxide Nanocomposites for Tissue Engineering. *ACS Appl. Mater. Inter.* **2016,** *8* (28), 17902. DOI: 10.1021/acsami.6b05635.

10. Wanger, C. D., *et al.*, *Handbook of X-ray Photoelectron Spectroscopy* Perkin-Elmer Corp., Physical Electronics Division: Eden Prairie, Minnesota, USA, 1979. 10.1002/sia.740030412

11. Wagner, C. D., *The NIST X-Ray Photoelectron Spectroscopy (XPS) Database*. U.S. Dept. of Commerce, National Institute of Standards and Technology: Gaithersburg, MD, USA, 1991.

12. ThermoFisher Oxygen X-ray photoelectron spectra. https://www.thermofisher.com/uk/en/home/materials-science/learning-center/periodic-table/non-metal/oxygen.html (accessed 8th June 2022).

13. Shchukarev, A. V.; Korolkov, D. V., XPS Study of group IA carbonates. *Cent. Eur. J. Chem.* **2004,** *2* (2), 347. DOI: 10.2478/bf02475578.

14. ThermoFisher Fluorine X-ray photoelectron spectra. https://www.thermofisher.com/uk/en/home/materials-science/learning-center/periodic-table/halogen/fluorine.html (accessed 8th June 2022).

15. *Standard Practice for Calculation of Corrosion Rates and Related Information from Electrochemical Measurements*. ASTM: West Conshohocken, PA 19428-2959, USA, 1999; Vol. ASTM G102-89(2015)e1.

16. Singh, B. P.; Nayak, S.; Nanda, K. K.; Jena, B. K.; Bhattacharjee, S.; Besra, L., The Production of a Corrosion Resistant Graphene Reinforced Composite Coating on Copper by Electrophoretic Deposition. *Carbon* **2013**, *61*, 47. DOI: 10.1016/j.carbon.2013.04.063.

17. Roberge, P. R., *Handbook of Corrosion Engineering*. McGraw-Hill Education:, New York, USA, **1999**.
